# Floristic Survey and Taxonomic Characteristics of Vascular Plants in Cerro Mohinora, Chihuahua, Mexico

**DOI:** 10.3390/plants15081267

**Published:** 2026-04-20

**Authors:** José Humberto Vega-Mares, Martín Martínez-Salvador, Ruben A. Martínez-Flores, Alicia Melgoza-Castillo

**Affiliations:** Facultad de Zootecnia y Ecología, Universidad Autónoma de Chihuahua (UACH), Perif. Francisco R. Almada Km 1, Chihuahua C.P. 31000, Mexico; jhvega@uach.mx (J.H.V.-M.); msalvador@uach.mx (M.M.-S.); p270838@uach.mx (R.A.M.-F.)

**Keywords:** protected natural area, microendemism, Sierra Madre Occidental

## Abstract

The Sierra Madre Occidental (SMO) is a vital biological corridor in Mexico, yet its floristic knowledge remains fragmented. This study inventories and analyzes the vascular flora of Cerro Mohinora, the highest peak in Chihuahua and a protected area critical for mountain biodiversity. Through the collection of 1200 specimens, we identified 350 taxa across 205 genera and 76 families. Asteraceae (57 taxa) and Poaceae (32) are the most species-rich, with hemicryptophytes dominating the life forms (55.7%). The native flora exhibits a predominantly Nearctic affinity (56.6%), followed by Neotropical (37.2%). Notably, 33.7% of species are endemic to Mexico, including 11.4% to the SMO and 2.9% related to microendemics. Cerro Mohinora serves as the type locality for 18 species, including *Salvia reginae*, which was discovered during the fieldwork of this study and formally described in 2019. It should be noted that not all of these taxa were recorded in the present survey. Regarding conservation, eight species are listed nationally and 54 internationally. Low exotic species representation (2.6%) contrasts with the vulnerability of the endemic flora. Our findings characterize Cerro Mohinora as a critical boreal refuge and an active evolutionary center, underscoring the need to strengthen conservation frameworks and management strategies to mitigate climate change impacts.

## 1. Introduction

The Sierra Madre Occidental (SMO) is the largest mountain system in Mexico and serves as a major biological corridor linking the temperate regions of northern Mexico with the tropical regions of the south [1,2]. Within the SMO in the state of Chihuahua, Cerro Mohinora represents the highest elevation and harbors high biodiversity across several taxonomic groups [3,4,5,6,7]. This is one of the reasons why the federal government designated this site as a Protected Natural Area [8]. However, the floristic inventory of the area remains incomplete. As part of a national program on taxonomic inventories, Cerro Mohinora was included to contribute to the development of a comprehensive floristic checklist for the state of Chihuahua [1,9].

The earliest floristic studies at Cerro Mohinora date back to 1898, when Edward W. Nelson and Edward A. Goldman conducted the first botanical explorations of the area. The collections made by these naturalists are housed at the Smithsonian Institution and resulted in eight new species being recorded [10]. Later, in 1959, Donovan S. Correll and Howard S. Gentry conducted botanical work at the site and documented four additional new species [11]. Other botanists, including Knobloch, Nesom, McDonald, González, and Peterson, have also conducted botanical collections in the area; however, floristic knowledge remains fragmented. A previous study [4] reported only 79 species for the summit of Cerro Mohinora and a similarity index of 7.2% with other high-mountain ecosystems in Mexico, indicating the presence of a highly distinctive flora. The lower and mid-elevations of the mountain are dominated by mixed conifer forests that include boreal relict elements of the genera *Picea*, *Pseudotsuga*, and *Abies*, associated with *Quercus* and *Pinus* [12,13].

Currently, the flora of Cerro Mohinora is exposed to multiple environmental pressures, including recurrent natural fires associated with global climatic variability. These events have locally manifested as reduced precipitation during the spring–summer period and increased frequency of summer droughts since the 1700s through the mid and late twentieth century [14,15]. In addition, livestock grazing and timber extraction, although subsistence-based and regulated under the management program due to its designation as a protected natural area [13], are not subject to continuous supervision.

High-elevation plant communities in the Sierra Madre Occidental, including those at Cerro Mohinora, are particularly vulnerable to climate change due to their orographic isolation and restricted altitudinal range. The distribution of these communities is largely controlled by environmental factors such as precipitation, temperature, continentality, and the presence of rocky outcrops [16]. Under current climate change scenarios, increasing temperatures and reduced moisture availability are expected to drive upward shifts in species distributions, potentially leading to changes in community composition [17]. These processes may alter ecosystem structure and functioning in the Sierra Madre Occidental [12], affecting phenological patterns and posing risks to the persistence of cold-adapted and endemic plant species [18,19].

This highlights the urgent need for comprehensive biological inventories to support effective planning and conservation of these fragile communities [12,16,20,21]. Based on the above considerations, the objective of this study was to document, identify, and analyze the vascular plant flora of Cerro Mohinora.

## 2. Results

### 2.1. Taxonomic Richness Composition

**Figure 1:**
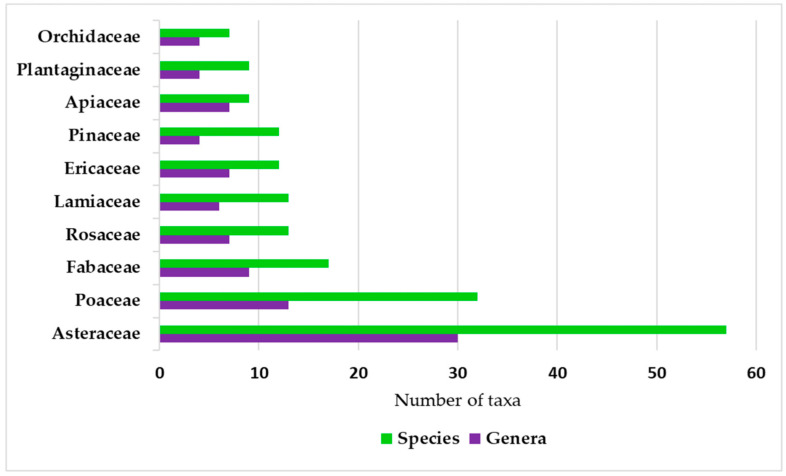
Species richness and relative representation of the predominant vascular plant families in Cerro Mohinora, Chihuahua, Mexico.

The Supplementary Materials (Table S1) provide a checklist of the flora of Cerro Mohinora, including information for each taxon on record source, life form, residency status, phytogeographic affinity, and conservation status (national and international) for each taxon. A total of 350 taxa were identified, distributed among 325 species and 25 infraspecific taxa. These were distributed among 205 genera and 76 families. Eudicots represented the most taxonomically diverse group, whereas lycophytes and magnoliids showed the lowest representation (Table 1). Species diversity is strongly concentrated in a relatively small number of families (Figure 1); the ten most species-rich families account for 51.7% of the total species richness and 44.3% of the generic diversity. Asteraceae is the most diverse family, accounting for 14.6% of the genera and 16.3% of the species, followed by Poaceae (6.3% and 9.1%) and Fabaceae (4.3% and 4.8%). At the generic level, the highest richness was recorded in *Muhlenbergia* (12 species), *Erigeron* (10), and *Pinus* (9); this last taxon constitutes the dominant structural component of the vegetation in the study area.

### 2.2. Flora Analysis

**Figure 2:**
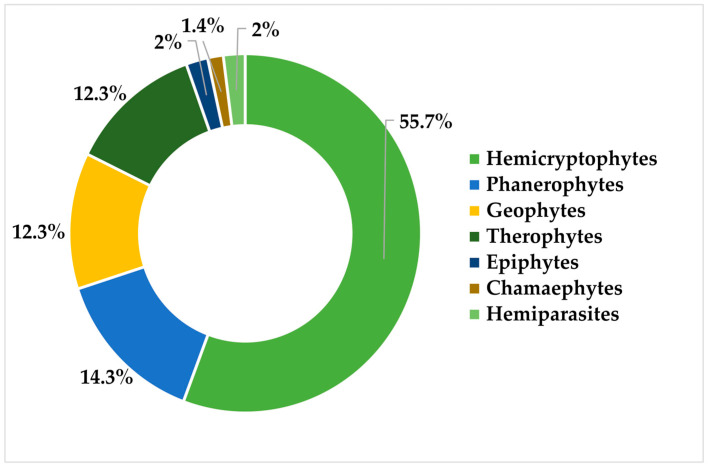
Biological spectrum of the vascular flora of Cerro Mohinora, Chihuahua, Mexico, based on the number of taxa. Life forms follow the Raunkiaer system [23] with the hierarchical scheme of [24].

**Figure 3:**
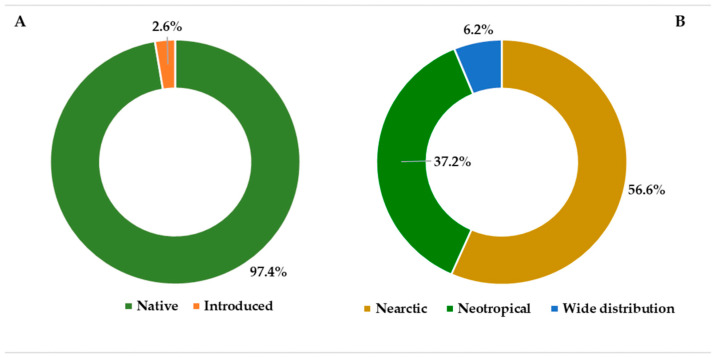
Composition of the vascular flora of Cerro Mohinora, Chihuahua, Mexico: (**A**) residency status of the total flora (*n* = 350 taxa), showing the proportion of native and introduced species; (**B**) phytogeographic affinities of the native flora (*n* = 341 taxa), categorized into Nearctic, Neotropical, and widely distributed lineages. Percentages are indicated for each category.

The biological spectrum of the vascular flora of Cerro Mohinora is dominated by hemicryptophytes, followed by phanerophytes and geophytes (Figure 2). A notable finding in the floristic composition is the low representation of vascular epiphytes, which account for only seven species and thus comprise a very small fraction of the total diversity. In terms of origin, native species represent the overwhelming majority of the flora, whereas introduced species account for only a small fraction (Figure 3A). Most introduced species recorded in the study area are widely distributed taxa of predominantly Eurasian or cosmopolitan origin, commonly associated with disturbed environments and human activities. Regarding phytogeographic affinity, Nearctic elements constitute the largest proportion of the flora (193 taxa), dominating both the forest canopy and summit communities (Figure 3B). Neotropical lineages follow in importance with 127 taxa, while the remaining flora consists of widely distributed elements (21 taxa).

**Figure 4:**
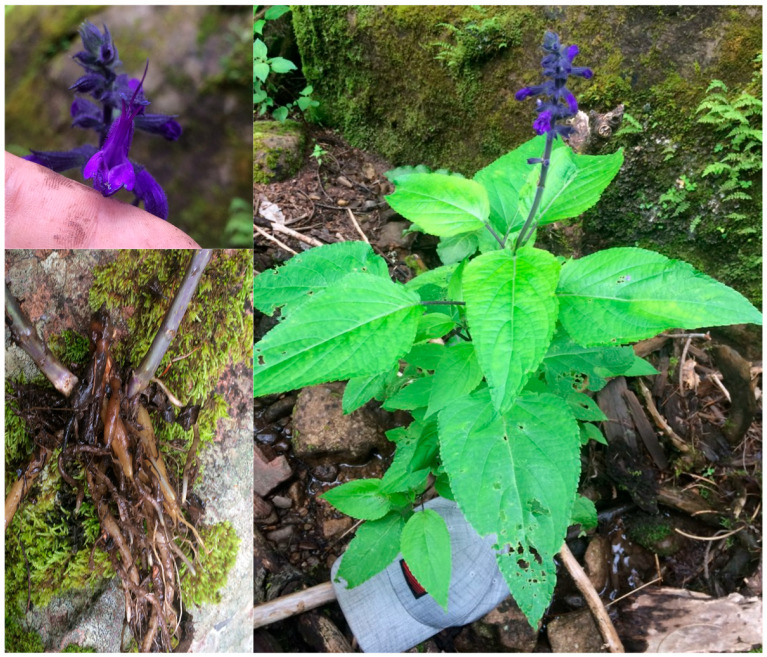
*Salvia reginae* J.G. González & J.H. Vega. Photograph of the holotype in situ at the type locality (Cerro Mohinora, Chihuahua, Mexico). The main panel shows the plant habit and flowering spike. Insets provide detailed views of the flower morphology (upper left) and the root system (lower left). Photo by J.H. Vega.

Endemic species represent a prominent component of the flora, with those restricted to Mexico accounting for 115 taxa. Regional endemism for the SMO represents 39 taxa, while local microendemics exclusive to Cerro Mohinora comprise 10 taxa (Table 2). This study resulted in the identification and formal description of *Salvia reginae* J.G. González & J.H. Vega [22], a species new to science whose type material was collected during the course of this project (Figure 4). This species belongs to the family Lamiaceae and represents a perennial geophyte characterized by a thick, tuberous root system that enables survival under harsh winter conditions. It exhibits a herbaceous to subshrub growth form, reaching 0.8–1.3 m in height. Leaves have long petioles (2.6–11 cm) and ovate–lanceolate blades measuring 8–23 cm in length and 4–11 cm in width, with a long-acuminate apex and serrate margins. The inflorescence consists of racemes 11–20 cm long bearing tubular flowers of intense blue to dark violet coloration, with pedicels 2.2–3.7 mm in length (Figure 4). In addition, 18 taxa have Cerro Mohinora as their type locality (locus typicus), as listed in Table 3.

**Table 2:** Levels of plant endemism and corresponding species richness in the flora of Cerro Mohinora, Chihuahua, Mexico.

Endemism Level	Number of Taxa	Percentage (%)	Source
Mexico	115	33.7	[9,25,26]
Sierra Madre Occidental	39	11.4	[27,28,29]
Chihuahua State	19	5.6	[9,26,27]
Cerro Mohinora (Local)	10	2.9	[27,28,29,30,31,32,33,34,35,36,37,38,39,40,41,42,43,44,45,46]

Although 47 species recorded in the study area have been assessed in the IUCN Red List (Table S1), the vast majority (44 species) fall within the Least Concern (LC) and Near Threatened (NT) categories. Therefore, our analysis focuses on taxa facing actual conservation risk (CR, EN, and VU) or those requiring legal protection under Mexican regulations (P, A, and Pr), thereby ensuring a more accurate interpretation of the conservation status of Cerro Mohinora. In this context, a total of 10 taxa were identified as having conservation status or legal protection (Table 4). According to the IUCN Red List, the species at greatest risk include *Picea mexicana* (EN), as well as *Juniperus blancoi* var. *huehuentensis* and *Agave bovicornuta* (VU). Additionally, eight species are listed in NOM-059-SEMARNAT-2010 [47], with *Picea mexicana* classified as Endangered (P).

It is important to note that several species protected at the national level, such as *Pseudotsuga menziesii* var. *glauca*, have not yet been evaluated (Not Evaluated, NE) under the IUCN framework. Furthermore, eight species recorded in the study area are included in Appendix II of CITES [48] (Table S1), indicating that their international trade should be regulated to avoid uses incompatible with their survival, although not all are classified as threatened under either the IUCN Red List or NOM-059.

**Table 3:** Vascular plant taxa with type localities at Cerro Mohinora, Chihuahua, Mexico (1898–2019).

Species (Taxa)	Collector/Coll. No./Herbarium [a]	Collected Year	Publication Year	Protologue	Recorded in This Study Yes/No
*Castilleja nelsonii* Eastw.	E.W. Nelson 4895 (US, GH)	1898	1909	[30]	No
*Gymnolomia serrata* B.L. Rob. & Greenm. (=*Verbesina scotiodonta* S.F. Blake)	E.W. Nelson 4891 (US)	1898	1899	[31]	No
*Penstemon mohinoranus* Straw	E.W. Nelson 4877 (US)	1898	1962	[32]	No
*Polemonium glabrum* J.F. Davidson	E.W. Nelson 4865 (US)	1898	1948	[33]	Yes
*Ranunculus gentryanus* L.D. Benson	E.W. Nelson 4888 (GH)	1898	1948	[34]	Yes
*Salvia muscarioides* Fernald	E.W. Nelson 4850 (US)	1898	1900	[35]	Yes
*Senecio mohinorensis* Greenm.	E.W. Nelson 4881 (GH)	1898	1907	[36]	Yes
*Zigadenus mohinorensis* Greenm. (=*Anticlea elegans* subsp. *elegans*)	E.W. Nelson 4875 (GH, US)	1898	1903	[37]	Yes
*Correllia montana* A.M. Powell [=*Galinsogeopsis montana* (A.M. Powell) Lichter-Marck]	D.S. Correll & Gentry 23185 (TEX)	1959	1973	[38]	Yes
*Romanschulzia correllii* Rollins	D.S. Correll & Gentry 23139 (LL)	1959	1984	[40]	No
*Stachys mohinora* B.L. Turner	D.S. Correll & Gentry 23181 (LL)	1959	1994	[39]	Yes
*Heliomeris multiflora* var. *macrocephala* Heiser (=*Heliomeris multiflora* var. *multiflora*)	Correll & Gentry 23170 (LL)	1959	1978	[41]	Yes
*Festuca diclina* Darbysh.	McDonald & Martinez 2392 (TEX)	1987	1995	[42]	No
*Erigeron caulinifolius* G.L. Nesom	G. Nesom 6483a (TEX)	1988	1989	[44]	Yes
*Erigeron mohinorensis* G.L. Nesom	G. Nesom 6448 (TEX)	1988	1989	[46]	Yes
*Erigeron macdonaldii* G.L. Nesom	McDonald & Nesom 2472 (TEX)	1988	1990	[45]	No
*Poa matri-occidentalis* subsp. *mohinorensis* Soreng & P.M. Peterson	Nesom 6475 & McDonald (TEX)	1988	2006	[43]	Yes
*Salvia reginae* J.G. González & J.H. Vega	H. Vega & S. Ochoa 3143 (CIDIIR)	2017	2019	[22]	Yes

Note. [a] Herbarium acronyms follow Index Herbariorum [49]. The location of the holotype is indicated in parentheses.

**Table 4:** Species of conservation concern in Cerro Mohinora, Chihuahua, Mexico, categorized by the IUCN Red List and the Mexican standard NOM-059-SEMARNAT-2010.

Taxa	Family	NOM-059-SEMARNAT-2010	IUCN Red List
*Agave bovicornuta* Gentry	Asparagaceae	--	VU
*Maianthemum racemosum* (L.) Link	Asparagaceae	A	NE
*Mammillaria senilis* Lodd. Ex Salm-Dyck	Cactaceae	A	LC
*Juniperus blancoi* var. *huehuentensis* R.P. Adams, S. González & M. González	Cupressaceae	--	VU
*Hesperocyparis lusitanica* (Mill.) Bartel	Cupressaceae	Pr	LC
*Monotropa hypopitys* L.	Ericaceae	Pr	NE
*Picea mexicana* Martínez	Pinaceae	P	EN
*Pinus durangensis* Martínez	Pinaceae	Pr	NT
*Pinus strobiformis* Engelm.	Pinaceae	Pr	LC
*Pseudotsuga menziesii* var. *glauca* (Beissn.) Franco	Pinaceae	Pr	NE

Note: NOM-059-SEMARNAT-2010 risk categories: P: Endangered; A: Threatened; Pr: Special Protection. IUCN Red List categories: EN: Endangered; VU: Vulnerable; NT: Near Threatened; LC: Least Concern; NE: Not Evaluated.

## 3. Discussion

### 3.1. Taxonomic Richness and Composition

The floristic richness recorded for Cerro Mohinora (350 taxa, 205 genera, and 76 families) is remarkably high given its relatively limited area of 5000 ha, particularly considering its altitudinal isolation. Compared with a previous study [4], which documented 79 taxa (62 genera and 33 families) within a 20 ha area restricted to the summit of Cerro Mohinora, the present study records an additional 271 taxa, 143 genera, and 43 families. However, this difference should be interpreted with caution, as the previous study focused on a limited high-elevation area, whereas the present study encompasses a broader altitudinal gradient and a much larger spatial extent. This increase in taxonomic representation can be attributed to the broader altitudinal gradient surveyed, which encompasses a greater diversity of microclimates and vegetation types that were not included in previous sampling efforts.

Although direct quantitative comparisons with adjacent mountain systems are limited by the scarcity of comprehensive and comparable floristic inventories in the region, the floristic richness recorded for Cerro Mohinora is consistent with patterns reported for the flora of Mexico [9], northern Mexico [1], and high-elevation summits of the Sierra Madre Occidental [4,12,16]. These ecosystems are typically characterized by high species turnover along altitudinal gradients and by the coexistence of Nearctic and Neotropical elements, which contribute to elevated levels of diversity. In this context, Cerro Mohinora stands out as a significant center of plant diversity, particularly considering its relatively small area and environmental heterogeneity. Future studies integrating standardized sampling across adjacent mountain systems would be valuable for assessing patterns of floristic similarity, beta diversity, and the influence of land use on species composition at the regional scale.

The taxonomic composition of the flora of Cerro Mohinora is dominated by the families Asteraceae, Poaceae, and Fabaceae. This pattern is consistent with that reported for the flora of Mexico [9], northern Mexico [1], and high-elevation summits of the SMO [4,12,16]. Genera such as *Muhlenbergia* (12 species), *Erigeron* (10 species), and *Pinus* (9 species) are particularly diverse, underscoring the importance of this site as a refuge for plant lineages adapted to high solar radiation and pronounced seasonal climatic variation [50]. Species of mixed conifer forests, which occupy only 0.3% of the SMO [12] and whose distribution includes Cerro Mohinora, define the physiognomic character of the vegetation through the presence of the gymnosperms *Abies* Martínez *durangensis*, *Pseudotsuga menziesii* var. *glauca* (Beissn.) Franco, and *Picea mexicana* Martínez, combined with *Pinus cooperi* C.E. Blanco, *P*. *leiophylla* var. *chihuahuana* (Engelm.) Shaw, *P*. *strobiformis* Engelm., *P*. *arizonica* Engelm., *P*. *teocote* Schiede ex Schltdl. & Cham., and *Juniperus deppeana* Steud. These species coexist with boreal relict elements such as *Quercus arizonica* Sarg., *Q*. *rugosa* Née, *Q*. *sideroxyla* Bonpl., *Arbutus bicolor* S. González, M. González & P.D. Sørensen, *A*. *xalapensis* Kunth, *A*. *tessellata* P.D. Sorensen, *Alnus oblongifolia* Torr., and *Cornus sericea* L. In the shrub layer occur *Arbutus occidentalis* McVaugh & Rosatti, *Ceanothus caeruleus* Lag., *Ribes ceriferum* Coville & Rose, *R*. *madrense* Coville & Rose, *Rubus idaeus* subsp. *strigosus* L. (Michx.) Focke, *R*. *pringlei* Rydb., and *Vaccinium caespitosum* Michx. In moist ravines located in the lower and middle portions of the mountain, *Hesperocyparis lusitanica* (Mill.) Bartel occurs, whereas in the upper zones, *Populus tremuloides* Michx., *Prunus serotina* var. *rufula* (Wooton & Standl.) McVaugh, and *P*. *serotina* var. *salicifolia* (Kunth) Koehne occur, giving the study area a complex vegetation structure. These species are consistent with those described for the vegetation of the SMO at elevations ranging from 2350 to nearly 3000 m [12,51]. Above 3200 m, the tree layer is replaced by mountain summit communities characterized by low-stature herbaceous vegetation adapted to harsh environmental conditions. Among the most frequent herbaceous taxa are *Alchemilla aphanoides* (Mutis ex L.f.) Rothm., *Castilleja patriotica* Fernald, *Draba helleriana* Greene, *Erigeron mohinorensis* G.L. Nesom, *Halenia recurva* (Sm.) C.K. Allen, *Packera scalaris* var. *scalaris* (Greene) C. Jeffrey, *P*. *umbraculifera* (S. Watson) W.A. Weber & Á.Löve, *Penstemon campanulatus* (Cav.) Willd., *Phacelia platycarpa* (Cav.) Spreng., *Ranunculus gentryanus* L.D. Benson, *Senecio mohinorensis* Greenm., and *Valeriana deltoidea* F.G. Mey. Grasses are represented by species such as *Bromus carinatus* var. *marginatus* (Nees ex Steud.) Barkworth, *B*. *richardsonii* Link, *Festuca diclina* Darbysh., and *Koeleria pyramidata* (Lam.) P. Beauv. Within the shrub layer, *Juniperus blancoi* var. *huehuentensis* R.P. Adams, S. González & M. González is the only species present in Cerro Mohinora, exhibiting a prostrate growth form [52] that represents a key morphological adaptation for reducing the effects of strong winds and high exposure typical of summit environments [16,53]. This relict species also occurs on the summits of Cerro Huehuento and Cerro Gordo [16], the highest peaks of the SMO in the state of Durango. Although some previous studies have classified the summit vegetation as alpine or subalpine [4,54], the strict altitudinal limits of alpine vegetation occur above 3650 m in northern Mexico [55] and above 4000 m in the central and southern regions of the country [56]. Because the maximum elevation of Cerro Mohinora is 3307 m, the vegetation is more accurately classified as a subalpine enclave or a mountain summit community [12,51]. The presence of these summit communities underscores the biological uniqueness of the site and its role as an active center of speciation, as evidenced by the concentration of local microendemics. It is important to note that some taxa for which Cerro Mohinora has been reported as the type locality were not recorded during the present survey. This absence may be related to factors such as limited detectability, seasonal variability, or changes in local environmental conditions and does not necessarily imply local extinction. The low proportion of exotic species (2.6%) of the total flora (nine species) reflects a relatively well-preserved ecosystem in which geographic and climatic barriers limit biological invasions. Nevertheless, the occurrence of *E*. *cicutarium*, *P*. *annua*, and *Rumex acetosella* L. on the mountain summit appears to be associated with tourist activity and vehicle traffic, highlighting the need for continuous monitoring. Such monitoring is critical to preventing additional introductions and limiting the potential spread of these species in high-elevation environments [57,58,59].

A temporal comparison with previous studies suggests a possible increase in the number of introduced species at the summit of Cerro Mohinora. McDonald et al. [4] reported only *Poa annua* during surveys conducted in 1987–1988, whereas the present study additionally recorded *Erodium cicutarium* and *Rumex acetosella* in the same high-elevation zone. Although this difference may reflect variations in sampling effort or spatial coverage, it could also indicate a gradual increase in the presence of non-native species over time. Notably, McDonald et al. [4] previously warned that infrastructure development, such as the construction of access roads, could facilitate the introduction and spread of exotic species. These observations highlight the importance of continued monitoring to assess potential changes in species composition in high-elevation environments.

### 3.2. Flora Analysis

With respect to the structure of this diversity, the pronounced dominance of hemicryptophytes (55.7%) in the biological spectrum of the flora of Cerro Mohinora represents a clear climatic signature of this mountain system (Figure 2), indicating adaptation to harsh winters and recurrent snowfall. In these plants, the perennating buds remain protected at or just below the soil surface, providing an important adaptive advantage in cold and strongly seasonal environments [23,60]. This pattern is consistent with observations from other mountain gradients, where the relative frequency of hemicryptophytes increases with elevation in response to declining temperatures and the persistence of snow cover [61,62,63]. Families such as Apiaceae (e.g., *Eryngium*) and Asteraceae (including *Ageratina*, *Erigeron*, *Packera*, and *Senecio*) represent prominent examples of hemicryptophytes. Phanerophytes (14.2%), which include trees and shrubs, are represented by families such as Cupressaceae (*Juniperus*), Ericaceae (*Arbutus*), Fagaceae (*Quercus*), Pinaceae (*Abies*, *Picea*, *Pinus*, and *Pseudotsuga*), and Rosaceae (*Prunus* and *Rubus*), which collectively form the forest canopy. Geophytes (12.2%) are particularly notable within Orchidaceae in this study, reflecting adaptations to growth cycles that include periods of dormancy.

The low abundance of epiphytic species in the study area can be attributed to both climatic and biological factors. The region is characterized by a pronounced dry season, particularly during late winter and early spring, which limits the moisture availability required for the establishment of epiphytic plants [56]. This condition helps explain why families such as Orchidaceae are represented exclusively by geophytic species in the study area, a strategy that allows them to avoid water stress and low temperatures [64].

Additionally, the dominance of coniferous species as phorophytes may further restrict the establishment of epiphytes, as these trees produce resins and other chemical compounds with allelopathic effects [65,66]. These factors likely limit the development of true epiphytic species and instead favor the prevalence of terrestrial plants with storage organs adapted to seasonal environmental stress.

The epiphytic flora of the study area is extremely limited and is restricted primarily to a small group of fern species with poikilohydric strategies, commonly referred to as “resurrection plants.” These species represent the only true epiphytes recorded (Table S1), highlighting the rarity of the epiphytic life form in Cerro Mohinora. Unlike most terrestrial herbaceous plants, which survive the dry season through underground storage organs or seasonal leaf loss, these ferns tolerate desiccation by entering a dormant state characterized by frond curling and biochemical adjustments that allow them to withstand extreme water loss [67]. This adaptation enables species such as *Pleopeltis polypodioides* (L.) E.G.Andrews & Windham to rapidly rehydrate and recover photosynthetic activity following the onset of seasonal moisture [68], allowing them to persist under the strongly seasonal climatic conditions of the region. This pattern contrasts with more humid tropical systems, where epiphytic diversity is typically much higher.

Phytogeographic analysis reveals a clear predominance of the Nearctic lineage (56.6%) within the native flora of Cerro Mohinora. This strong Holarctic influence is consistent with the high elevation and cool temperate climate of the area, reinforcing the role of Cerro Mohinora as a critical boreal refuge for northern plant lineages [12,69,70]. As the highest peak in the state of Chihuahua and the second highest in the SMO [16], this mountain massif functions as a center of isolation and persistence for relict taxa following the Pleistocene glacial cycles [12,71,72].

In contrast, the substantial Neotropical component (37.2%) highlights the role of the SMO as a major biological corridor where temperate northern species converge with lineages of montane–austral origin from the south. The coexistence of elements from different biogeographic kingdoms further supports the recognition of Cerro Mohinora as a key nodal site within the Mexican Transition Zone. The category of widely distributed taxa (6.2%), which includes American transitional elements, further complements this biogeographic pattern. Cerro Mohinora represents a notable center of endemism, where the presence of local microendemism (2.9%; 10 taxa) restricted exclusively to the site is particularly remarkable. The strong climatic and geographic specialization of these taxa substantially increases their vulnerability, especially considering that approximately 58% of endemic plant species worldwide are already regarded as extinct or threatened [73]. The local microendemics recorded in the study area include *Erigeron caulinifolius* G.L. Nesom, *E*. *macdonaldii* G.L. Nesom, *E*. *mohinorensis*, *S*. *mohinorensis*, *Verbesina scotiodonta* S.F. Blake, *Romanschulzia correllii* Rollins, *Stachys mohinora* B.L. Turner, *Penstemon mohinoranus* Straw, *Poa matri-occidentalis* subsp. *mohinorensis* Soreng & P.M. Peterson, and *Polemonium glabrum* J.F. Davidson. This high level of endemism, at both regional and local scales, supports the recognition of Cerro Mohinora as an active center of speciation and in situ evolutionary processes.

The description of *Salvia reginae* [22] as a species new to science highlights the potential for additional taxonomic discoveries in the area. In total, Cerro Mohinora represents the type locality for 18 taxa (Table 3). The rediscovery of most of these historical type taxa supports the long-term ecological stability of the area. However, the absence of some specimens suggests the possible existence of highly localized and fragile populations that may be particularly vulnerable to disturbance [73,74,75]. The conservation status of the flora of Cerro Mohinora reveals a marked disparity between the biological uniqueness of the area and its current legal protection framework. A total of 47 species recorded in the study area are included in the IUCN Red List, whereas only eight taxa are listed under risk categories in the Mexican standard NOM-059-SEMARNAT-2010. However, it is important to note that the majority of species have not yet been evaluated (Not Evaluated, NE) under the IUCN Red List framework and are therefore not represented in these categories. Among the IUCN-listed species, the presence of *P*. *mexicana*, classified as Endangered (EN), is of particular concern. In addition, *Agave bovicornuta* Gentry and *Juniperus blancoi* var. *huehuentensis* are listed as Vulnerable (VU), while *Quercus mcvaughii* Spellenb., *Pinus durangensis* Martínez, and *P*. *lumholtzii* B.L. Rob. & Fernald are categorized as Near Threatened (NT). Furthermore, the nine species included in Appendix II of CITES—primarily orchids (*Dichromanthus aurantiacus* (Lex.) Salazar & Soto Arenas, *D*. *michuacanus* (Lex.) Salazar & Soto Arenas, *Malaxis macrostachya* (Lex.) Kuntze, *M*. *novogaliciana* R. González ex McVaugh, *Microthelys rubrocalosa* (B.L. Rob. & Greenm.) Garay, *Platanthera brevifolia* (Greene) Kraenzl., *Tamayorkis porphyrea* (Ridl.) Salazar & Soto Arenas), and two cactus species (*Mammillaria senilis* Lodd. Ex Salm-Dyck and *Opuntia robusta* H.L. Wendl. ex Pfeiff.)—complement national and global protection frameworks. This alignment highlights their susceptibility to international trade and reinforces the need for strict regulations to ensure their long-term survival in the study area.

At the national level, NOM-059-SEMARNAT-2010 lists *Picea mexicana* Martínez as Endangered (P); *Maianthemum racemosum* (L.) Link and *Mammillaria senilis* as Threatened (A); and *Hesperocyparis lusitanica*, *Monotropa hypopitys*, *Pinus durangensis*, *Pinus strobiformis*, and *Pseudotsuga menziesii* var. *glauca* under Special Protection (Pr). The inclusion of *Mammillaria senilis* in NOM-059-SEMARNAT-2010, the IUCN Red List, and CITES, as well as the presence of several pine and other conifer species listed in two of these protection frameworks, underscores the need for their conservation. The marked disparity between the high number of species considered vulnerable at the global level (47 listed by the IUCN) and those legally protected in Mexico (8 taxa included in NOM-059-SEMARNAT-2010) highlights the urgent need to update and strengthen national conservation listings [76,77,78]. The designation of Cerro Mohinora as a Flora and Fauna Protection Area (APFF) represents a crucial step for its conservation. Nevertheless, the fragility of its endemic flora in the face of climate change, together with pressures such as livestock grazing and recurrent forest fires [15,54], highlights the need for strengthened management and conservation efforts. Considering that ecosystem services provided by Protected Natural Areas in Mexico have been valued at approximately USD 391 billion [79], while another study [80] estimated a willingness to pay of USD 90.48 per visitor to access Cerro Mohinora, contingent upon investment in conservation measures.

Despite the substantial increase in floristic knowledge achieved in this study, several limitations should be acknowledged. This work represents a floristic inventory developed within the framework of the management plan of the Cerro Mohinora Protected Natural Area [13]. Although previous studies reported only 79 species within a restricted area of approximately 20 ha, the present study documents 350 taxa across a broader area of 5000 ha. However, the total extent of the protected area exceeds 255,000 ha, indicating that a significant portion of the territory remains unexplored and that the current inventory likely underestimates the total floristic richness.

Additional limitations include the large spatial extent and complex topography of the area, limited financial resources for extensive field campaigns, and current security constraints that restrict access to certain zones. Furthermore, anthropogenic pressures such as overgrazing, land-use change, altered fire regimes, and deforestation [13,14,54] pose ongoing threats to biodiversity. At the same time, the high elevation and rugged terrain of Cerro Mohinora have allowed for the persistence of refugial habitats that harbor unique plant assemblages. However, these environments are particularly vulnerable to climate change. Future research should prioritize expanding spatial coverage, implementing long-term monitoring programs, and applying standardized sampling approaches to better estimate total species richness, assess beta diversity, and support conservation and management strategies in this high-mountain ecosystem.

## 4. Materials and Methods

### 4.1. Study Area

**Figure 5:**
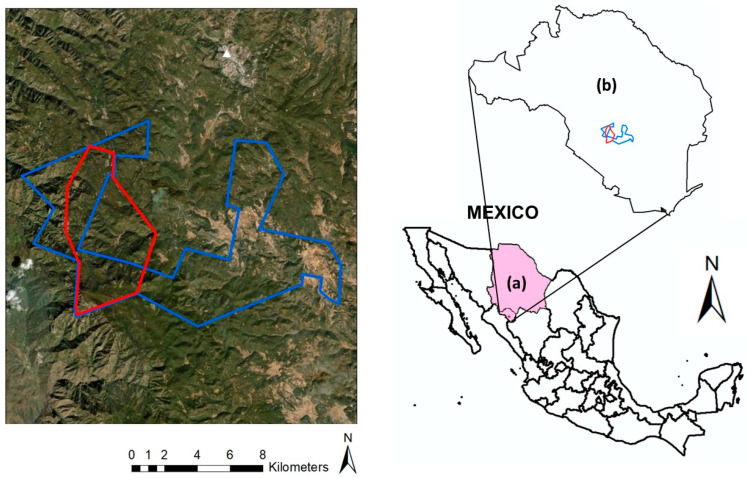
Location of the study area at Cerro Mohinora, Chihuahua, Mexico. Panel (**a**) shows the location of the state of Chihuahua within Mexico, panel (**b**) depicts the municipality of Guadalupe y Calvo. In the left panel, the red polygon delineates the study area, while the light blue line represents the boundary of the Flora and Fauna Protection Area (APFF).

Cerro Mohinora is located in southwestern Chihuahua, Mexico, in the municipality of Guadalupe y Calvo, within the biogeographic province of the SMO [70,81]. The area borders the state of Durango to the southeast and Sinaloa to the southwest. Its geographic limits range from 25°56′39″ N, 106°58′46″ W to 26°00′18″ N, 106°59′53″ W (Figure 5). The study area covers approximately 5000 ha, representing 55% of the designated APFF.

The climate of Cerro Mohinora is characterized by a mean annual temperature ranging from 10 °C to 12 °C, making it one of the coldest regions in Chihuahua due to its high elevation [13,82]. Annual precipitation ranges between 800 and 1200 mm [15], with a strongly seasonal pattern, concentrated from July to September under the influence of the Mexican monsoon, while the dry season is most pronounced in March and April [82].

The terrain is predominantly rugged, with slopes ranging from 0.5% on small plateaus to as much as 80% on steep mountain slopes [13]. Geologically, the mountain is composed of extrusive igneous rocks (ignimbrites and rhyolitic tuffs) of Oligocene–Miocene age [83]. Over these substrates, Haplic Phaeozems occur in the lower elevations, whereas Lithosols dominate the higher zones [84]. The altitudinal gradient ranges from 2350 to 3307 m a.s.l. Climate conditions vary from semi-arid, semi-cold [BS1k″w] in the lower elevations to temperate subhumid [C(w2)x′] at the summit, with mean annual precipitation ranging from 800 to 1200 mm [82,85,86]. Biogeographically, the site lies within the SMO province [87]. The predominant vegetation consists of mixed conifer forest, with a canopy dominated by *Abies durangensis* Martínez, *P*. *menziesii* var. *glauca*, and several species of *Pinus* and *Quercus* [12,13]. Above 3200 m, the tree layer is replaced by a mountain summit community with subalpine physiognomic affinity, characterized by prostrate shrubs such as *J*. *blancoi* var. *huehuentensis* and herbaceous assemblages [1,4,13].

### 4.2. Field and Laboratory Work

Field collections were conducted between July 2017 and December 2018, encompassing all four seasons of the year. Specimens were collected under permit FUAT-019 issued by the Secretaría de Medio Ambiente y Recursos Naturales (SEMARNAT), which authorized the collection of multiple taxonomic groups, including plants.

**Figure 6:**
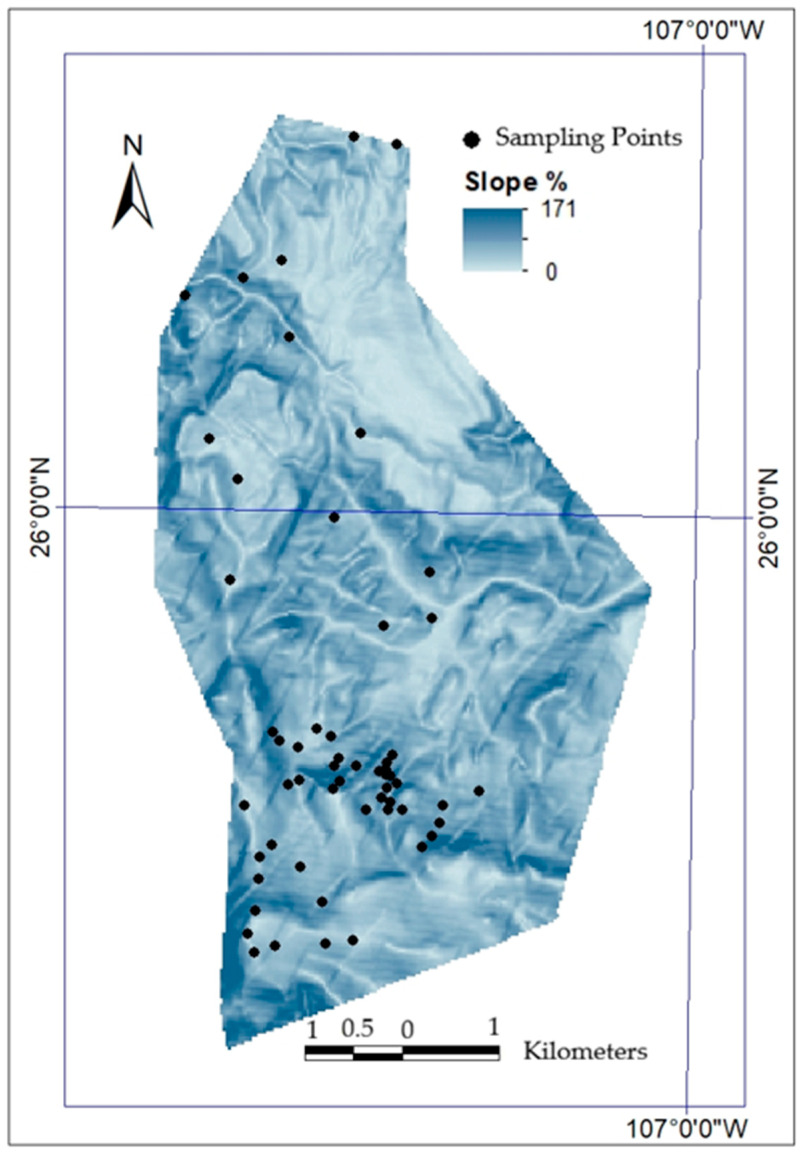
Spatial distribution of georeferenced sampling points across the study area in Cerro Mohinora, Chihuahua, Mexico, illustrating the coverage of topographic heterogeneity (slope) and environmental gradients sampled during the floristic survey.

Sampling followed an intensive floristic survey approach based on directed exploratory walks rather than fixed transects. Exhaustive collections were carried out to capture the topographic and vegetation heterogeneity of the study area over 30 sampling days, resulting in 58 georeferenced collection points (Figure 6). Sampling was distributed across the main environmental gradients, including variation in altitude and vegetation types, in order to ensure representative coverage of the study area.

**Figure 7:**
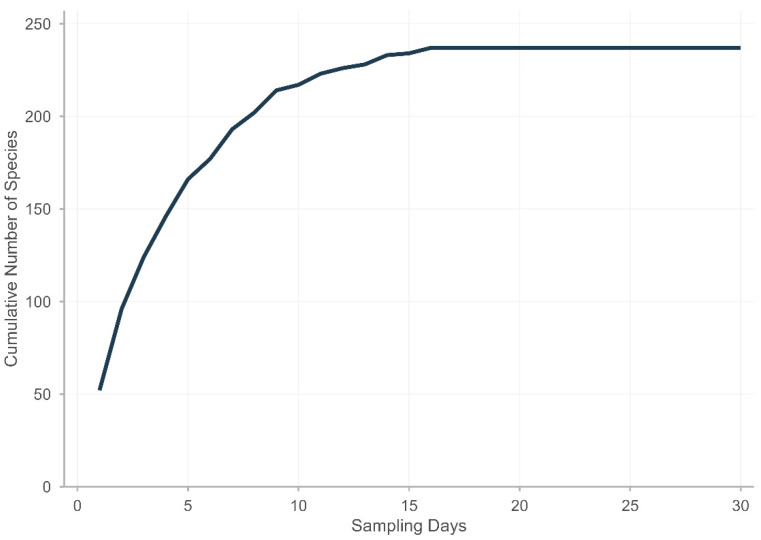
Species accumulation curve based on direct sampling effort (30 days of field collection) at Cerro Mohinora, Chihuahua, Mexico.

Sampling representativeness was evaluated using a species accumulation curve [88], with sampling units defined as collection days (*n* = 30). The species accumulation curve is presented in Figure 7. Additionally, the non-parametric estimator Chao2 was calculated to estimate potential species richness using the statistical software R (version 4.5.1) [89] within the RStudio (version 2025.9.1.401) environment [90].

A total of 1200 specimens were collected and processed following standard herbarium techniques [91,92]. Taxonomic identification was based on morphological examination and the use of specialized taxonomic keys [93,94,95,96,97]. For type taxa, taxa with complex identities, and topotypes, original protologues were consulted, and type specimens were examined through digital platforms such as Global Plants (JSTOR) [98] and the National Museum of Natural History (US) [99]. Additional verification was provided by specialists from the CIIDIR herbarium for selected taxonomic groups. To complement the information on species presence and distribution, previous botanical explorations and floristic inventories from Cerro Mohinora were reviewed [4,10,11]. Floristic treatments from neighboring regions were also reviewed to identify potential new records for the study area [12,16,100,101]. In addition, databases such as SEINet [29] and the Mexican Herbarium Network [28] online were consulted, along with virtual herbarium collections. Particular attention was given to the US herbarium, which houses the earliest collections from Cerro Mohinora, as well as to other major herbaria, including MEXU, TEX, LL, ARIZ, and NY. Herbarium acronyms follow Index Herbariorum online [49]. Nomenclature, taxonomic acceptance, and authorities were standardized according to Tropicos [26] and POWO [25]. Family circumscription and classification followed PPG I [102] for ferns, [103] for gymnosperms, and APG IV [104] for angiosperms. Voucher specimens were deposited in the UACH-HER herbarium, with duplicates housed at the CIIDIR-Durango herbarium.

Based on field observations and notes, specimens were classified according to the life-form system [23], following the hierarchical scheme adapted by [24]. Species were categorized as native or introduced according to the criteria of [9] and the Weeds of Mexico portal [105]. The phytogeographic affinity of native species within the Mexican Transition Zone was analyzed by assigning lineages to three categories: (1) Nearctic (northern temperate elements), (2) Neotropical (montane–austral elements), and (3) widely distributed taxa. This classification follows the centers of diversification proposed by [70,106]. Endemism was analyzed hierarchically across four levels of geographic restriction: national (Mexico), regional (SMO), state (Chihuahua), and local (microendemism restricted to Cerro Mohinora). Endemism and type localities were verified through consultation of original protologues and global databases POWO and JSTOR Global Plants. Conservation status was subsequently assessed by cross-checking species against NOM-059-SEMARNAT-2010 [106], the IUCN Red List, and the CITES Appendices.

## 5. Conclusions

The vascular flora of Cerro Mohinora exhibits exceptional taxonomic richness, acting as a strategic boreal refuge and a critical node within the Mexican Transition Zone. The dominance of hemicryptophytes and the prevalence of Nearctic lineages underscore a strong climatic specialization to high-altitude environments. Taxonomic richness, the local microendemics, and 18 type localities indicate that this site functions as an active center of speciation and evolutionary persistence. However, the marked disparity between global vulnerability assessments and national protection frameworks, coupled with anthropogenic pressures and climate change, requires conservation priority. These findings provide a fundamental baseline for strengthening the management of this high-elevation subalpine enclave.

## Figures and Tables

**Table 1:** Taxonomic composition of the vascular flora of Cerro Mohinora, Guadalupe y Calvo, Chihuahua, Mexico.

Taxonomic Groups	Number of Families	Number of Genera	Number of Species	Number of Infraspecific Taxa
Lycophytes	1	1	1	0
Ferns	10	15	19	2
Gymnosperms (Piniidae)	2	6	13	3
Angiosperms				
Magnoliids	1	1	1	0
Monocots	10	34	60	3
Eudicots	52	148	231	17
Total	76	205	325	25

## Data Availability

Data are contained within the article and Supplementary Materials.

## Supplementary Materials

The following supporting information can be downloaded at: https://www.mdpi.com/article/10.3390/plants15081267/s1, Table S1: Checklist of vascular plant species of Cerro Mohinora, Chihuahua, Mexico.
