# Supplementary material for: Floristic Survey and Taxonomic Characteristics of Vascular Plants in Cerro Mohinora, Chihuahua, Mexico
Source: Plants (Basel). 2026 Apr 20;15(8):1267. doi: 10.3390/plants15081267 (PMC13120365; doi:10.3390/plants15081267)
Supplement: Supplementary file 1 [file plants-15-01267-s001.zip › plants-4243980-supplementary.pdf]

Supplementary Material

Table S1. Checklist of vascular plant species of Cerro Mohinora, Chihuahua, Mexico. The taxonomic classification and arrangement of families followed the [102] system for ferns, [103] for gymnosperms, and [104] for angiosperms. Within each major group, families and species were listed alphabetically.

Record source: CS: Current study; OS: Other sources

Life cycle: A: annual; P: perennial.

Residency status: N: native; I: introduced.

Life forms (Raunkiaer): H: hemicryptophyte; F: phanerophyte; G: geophyte; T: therophyte; E: epiphyte; Ca: chamaephyte; Hp: Hemiparasites

Phytogeographic affinity (Phyt. af): NEA: Nearctic; NEO: Neotropical; AD: wide distribution; ---: not applicable (for introduced species).

Endemism: EM: endemic to Mexico; ESMO: endemic to the Sierra Madre Occidental; ECH: endemic to the state of Chihuahua; EMO: microendemic to Cerro Mohinora (local scale); ---: Not Applicable

Conservation status (NOM-059-SEMARNAT-2010): Pr: special protection; A: threatened; P: endangered; ---: Not Applicable

IUCN Red List categories: LC: least concern; NT: near threatened; VU: vulnerable; EN: endangered; CR: critically endangered.

CITES Appendices: II: species not necessarily threatened with extinction but subject to controlled trade.

GROUP/FAMILY	Taxa	Record source	Life Cycle	Residency status	Life form	Phyt. af.	Endemism	Conservation status
**I. LYCOPHYTES**								---
Selaginellaceae	*Selaginella underwoodii* Hieron.	CS	P	N	H	NEO	---	---
**II. FERNS**								
Aspleniaceae	*Asplenium monanthes* L.	CS	P	N	H	WD	---	---
Aspleniaceae	*Thelypteris pilosa* (M.Martens & Galeotti) Crawford	OS	P	N	H	NEO	---	---
Aspleniaceae	*Woodsia mexicana* Fée	OS	P	N	H	NEA	---	---
Aspleniaceae	*Woodsia phillipsii* Windham	CS	P	N	H	NEA	---	---
Athyriaceae	*Athyrium filix-femina* (L.) Roth	CS	P	N	H	WD	---	---
Blechnaceae	*Blechnum ghiesbreghtii* (Baker) C.Chr.	OS	P	N	H	NEO	---	---
Cystopteridaceae	*Cystopteris fragilis* (L.) Bernh.	CS	P	N	G	WD	---	---
Dryopteridaceae	*Dryopteris cinnamomea* (Cav.) C. Chr.	OS	P	N	H	NEA	---	---

GROUP/FAMILY	Taxa	Record source	Life Cycle	Residency status	Life form	Phyt. af.	Endemism	Conservation status
Dryopteridaceae	*Dryopteris wallichiana* subsp. *wallichiana (Spreng.) Hyl.*	CS	P	N	H	WD	---	---
Hymenophyllaceae	*Hymenophyllum tunbrigense* (L.) Sm.	OS	P	N	E	WD	---	---
Hymenophyllaceae	*Trichomanes radicans* Sw.	OS	P	N	H	WD	---	---
Ophioglossaceae	*Botrychium schaffneri* Underw.	CS	P	N	G	NEA	---	---
Plagiogyriaceae	*Plagiogyria pectinata* (Liebm.) Lellinger	OS	P	N	H	NEO	---	---
Polypodiaceae	*Pecluma hartwegiana* (Hook.) F.C.Assis & Salino	OS	P	N	E	NEO	---	---
Polypodiaceae	*Pleopeltis guttata* (Maxon) E.G. Andrews & Windham	OS	P	N	E	NEO	EM	---
Polypodiaceae	*Pleopeltis polylepis* (Roem. Ex Kunze) T. Moore		P	N	E	NEO	---	---
Polypodiaceae	*Pleopeltis polylepis* var. *erythrolepis* (Weath.) T.Wendt	OS	P	N	E	NEO	EM	---
Polypodiaceae	*Pleopeltis polypodioides* (L.) E. G. Andrews & Windham	CS	P	N	E	WD	---	---
Polypodiaceae	*Polypodium hesperium* Maxon	OS	P	N	E	NEA	---	---
Pteridaceae	*Hemionitis arizonica* (Maxon) Christenh.	OS	P	N	H	NEO	---	---
Pteridaceae	*Hemionitis hirsuta* (Link) Christenh.		P	N	H	NEO	---	---
**III. Gymnosperms (PINIIDAE)**								
Cupressaceae	*Hesperocyparis lusitanica* (Mill.) Bartel	CS	P	N	F	NEA	---	NOM-Pr, IUCN-LC
Cupressaceae	*Juniperus blancoi* var. *huehuentensis* R.P. Adams, S. González & M. González	CS	P	N	F	NEA	EM, ESMO	IUCN-VU
Cupressaceae	*Juniperus deppeana* Steud.	CS	P	N	F	NEA	---	---
Cupressaceae	*Juniperus durangensis* Martínez	CS	P	N	F	NEA	EM	IUCN-LC
Pinaceae	*Abies durangensis* Martínez	CS	P	N	F	NEA	EM	IUCN-LC
Pinaceae	*Picea mexicana* Martínez	CS	P	N	F	NEA	EM	NOM-P, IUCN-EN
Pinaceae	*Pinus arizonica* Engelm.	OS	P	N	F	NEA	---	IUCN-LC
Pinaceae	*Pinus cooperi* C.E. Blanco	OS	P	N	F	NEA	EM, ESMO	---
Pinaceae	*Pinus durangensis* Martínez	CS	P	N	F	NEA	EM	NOM-Pr, IUCN-NT

GROUP/FAMILY	Taxa	Record source	Life Cycle	Residency status	Life form	Phyt. af.	Endemism	Conservation status
Pinaceae	*Pinus engelmannii* Carrière	OS	P	N	F	NEA	---	IUCN-LC
Pinaceae	*Pinus hartwegii* Lindl.	OS	P	N	F	NEA	---	IUCN-LC
Pinaceae	*Pinus leiophylla* var. *chihuahuana* (Engelm.) Shaw	OS	P	N	F	NEA	---	IUCN-LC
Pinaceae	*Pinus lumholtzii* B. L. Rob. & Fernald	CS	P	N	F	NEA	EM	IUCN-NT
Pinaceae	*Pinus strobiformis* Engelm.	CS	P	N	F	NEA	---	NOM-Pr, IUCN-LC
Pinaceae	*Pinus teocote* Schiede ex Schltdl. & Cham.	OS	P	N	F	NEA	---	IUCN-LC
Pinaceae	*Pseudotsuga menziesii* var. *glauca* (Beissn.) Franco	CS	P	N	F	NEA	---	NOM-Pr
**IV. ANGIOSPERMS**								
**IV.I Magnoliids**								
Piperaceae	*Peperomia bracteata* A.W.Hill	CS	P	N	H	NEO	---	---
**IV.II. Monocots**		CS					---	---
Asparagaceae	*Agave bovicornuta* Gentry	CS	P	N	H	NEO	EM	IUCN-VU
Asparagaceae	*Echeandia longipedicellata* Cruden	CS	P	N	G	NEO	---	---
Asparagaceae	*Maianthemum racemosum* (L.) Link	CS	P	N	H	NEO	---	NOM-A
Asparagaceae	*Manfreda singuliflora* (S. Watson) Rose	CS	P	N	G	NEO	EM	---
Asparagaceae	*Milla biflora* Cav.	CS	P	N	G	NEO	---	---
Commelinaceae	*Commelina tuberosa* L.	OS	P	N	G	NEO	---	---
Commelinaceae	*Tradescantia pygmaea* D.R. Hunt	CS	P	N	G	NEO	EM, ESMO	---
Cyperaceae	*Carex chihuahuensis* Mack.	CS	P	N	H	NEA	---	---
Cyperaceae	*Carex longicaulis* Boeckeler	OS	P	N	G	NEA	EM	---
Cyperaceae	*Cyperus hypopitys* G.C. Tucker	OS	P	N	H	NEO	---	---
Cyperaceae	*Cyperus fendlerianus* Boeckeler	OS	P	N	H	NEA	---	---
Cyperaceae	*Cyperus seslerioides* Kunth	CS	P	N	H	NEO	---	---
Cyperaceae	*Cyperus sphaerolepis* Boeckeler	CS	P	N	G	NEA	---	---
Cyperaceae	*Cyperus pennellii* O'Neill & Ben. Ayers	CS	P	N	H	NEO	---	---
Hypoxidaceae	*Hypoxis mexicana* Schult. & Schult.f.	CS	P	N	G	NEO	---	---
Iridaceae	*Nemastylis tenuis* (Herb.) Benth. & Hook. f. ex S. Watson	OS	P	N	G	NEO	---	---
Iridaceae	*Sisyrinchium convolutum* (Ker Gawl.) W.T.Aiton	OS	P	N	H	NEO	---	---

GROUP/FAMILY	Taxa	Record source	Life Cycle	Residency status	Life form	Phyt. af.	Endemism	Conservation status
Juncaceae	*Luzula caricina* E. Mey.	CS	P	N	H	NEA	---	---
Juncaceae	*Luzula comosa* E. Mey.	CS	P	N	H	NEA	---	---
Juncaceae	*Luzula parviflora* (Ehrh.) Desv.	CS	P	N	H	WD	---	---
Liliaceae	*Calochortus ownbeyi* M.A. García-Mart., Aaron Rodr. & H.P.McDonald	CS	P	N	G	NEA	EM	---
Liliaceae	*Calochortus venustulus* Greene	CS	P	N	G	NEA	EM	---
Melanthiaceae	*Anticlea elegans* (Pursh) Rydb.	CS	P	N	G	NEA	---	---
Melanthiaceae	*Schoenocaulon megarrhizum* M.E. Jones	CS	P	N	G	NEA	EM	---
Orchidaceae	*Dichromanthus aurantiacus* (Lex.) Salazar & Soto Arenas	CS	P	N	G	NEO	---	CITES-II
Orchidaceae	*Dichromanthus michuacanus* (Lex.) Salazar & Soto Arenas	CS	P	N	G	NEO	---	CITES-II
Orchidaceae	*Malaxis macrostachya* (Lex.) Kuntze	CS	P	N	G	NEO	---	CITES-II
Orchidaceae	*Malaxis novogaliciana* R. González ex McVaugh	CS	P	N	G	NEO	---	CITES-II
Orchidaceae	*Microthelys rubrocalosa* (B.L.Rob. & Greenm.) Garay	CS	P	N	G	NEO	---	CITES-II
Orchidaceae	*Platanthera brevifolia* (Greene) Kraenzl.	CS	P	N	G	NEA	---	CITES-II
Orchidaceae	*Tamayorkis porphyrea* (Ridl.) Salazar & Soto Arenas	OS	P	N	G	NEO	---	CITES-II
Poaceae	*Agrostis scabra* Willd.	OS	P	N	H	NEA	---	---
Poaceae	*Avenella flexuosa* (L.) Drejer	OS	P	N	H	WD	---	---
Poaceae	*Bromus anomalus* Rupr. ex E. Fourn.	CS	P	N	H	NEA	---	---
Poaceae	*Bromus carinatus* var. *marginatus* (Nees ex Steud.) Barkworth	OS	P	N	H	NEA	---	---
Poaceae	*Bromus richardsonii* Link	OS	P	N	H	NEA	---	---
Poaceae	*Chloris submutica* Kunth	CS	P	N	H	NEO	---	---
Poaceae	*Elymus arizonicus* (Scribn. & J.G.Sm.) Gould	CS	P	N	H	NEA	---	---
Poaceae	*Festuca diclina* Darbysh.	OS	P	N	H	NEA	EM, ESMO, ECH	---
Poaceae	*Festuca pringlei* St.-Yves	OS	P	N	H	NEA	EM	---
Poaceae	*Festuca rubra* L.		P	N	H	WD	---	IUCN-LC
Poaceae	*Koeleria pyramidata* (Lam.) P. Beauv.	OS	P	N	H	WD	---	---

GROUP/FAMILY	Taxa	Record source	Life Cycle	Residency status	Life form	Phyt. af.	Endemism	Conservation status
Poaceae	*Muhlenbergia cenchroides* (Humb. & Bonpl. ex Willd.) P.M. Peterson	CS	A	N	T	NEO	---	---
Poaceae	*Muhlenbergia emersleyi* Vasey	CS	P	N	H	NEO	---	---
Poaceae	*Muhlenbergia montana* (Nutt.) Hitchc.	CS	P	N	H	NEA	---	---
Poaceae	*Muhlenbergia mucronata* (Kunth) Trin.	CS	P	N	H	NEO	EM	---
Poaceae	*Muhlenbergia pectinata* Goodd.	OS	A	N	T	NEO	---	---
Poaceae	*Muhlenbergia quadridentata* (Kunth) Trin.	OS	P	N	H	NEO	---	---
Poaceae	*Muhlenbergia ramulosa* (Kunth) Swallen	OS	A	N	T	NEO	---	---
Poaceae	*Muhlenbergia rigida* (Kunth) Kunth	CS	P	N	H	NEO	---	---
Poaceae	*Muhlenbergia schmitzii* Hack.	OS	A	N	T	NEO	EM	---
Poaceae	*Muhlenbergia uniseta* (Lag.) Columbus	CS	A	N	T	NEO	EM	---
Poaceae	*Muhlenbergia vaginata* Swallen	OS	A	N	T	NEO	---	---
Poaceae	*Muhlenbergia virescens* (Kunth) Kunth	CS	P	N	H	NEA	---	---
Poaceae	*Paspalum prostratum* var. *pygmaeum* Scribn. & Merr.	OS	A	N	T	NEO	---	---
Poaceae	*Peyritschia deyeuxioides* (Kunth) Finot	CS	P	N	H	NEO	---	---
Poaceae	*Peyritschia filifolia* (Scribn. ex Beal) P.M.Peterson, Soreng, Romasch. & Barberá	CS	P	N	H	NEO	EM, ESMO	---
Poaceae	*Poa annua* L.	CS	A	I	T	-----	---	IUCN-LC
Poaceae	*Poa fendleriana* (Steud.) Vasey	CS	P	N	H	NEA	---	---
Poaceae	*Poa matris-occidentalis* subsp. *mohinorensis* Soreng & P.M.Peterson	CS	P	N	H	NEA	EM, ESMO, ECH, EMO	---
Poaceae	*Poa strictiramea* Hitchc.	CS	P	N	H	NEA	---	---
Poaceae	*Sporobolus indicus* (L.) R. Br.	CS	P	I	H	-----	---	IUCN-LC
Poaceae	*Zuloagaea bulbosa* (Kunth) Bess.	CS	P	N	G	NEO	---	---
**IV.II. Eudicots**								
Amaranthaceae	*Dysphania incisa* (Poir.)	CS	A	N	T	NEA	---	---
Apiaceae	*Conioselinum mexicanum* J.M.Coult. & Rose	OS	P	N	H	NEA	---	---
Apiaceae	*Donnellsmithia ternata* (S. Watson) Mathias & Costance	CS	P	N	H	NEA	EM	---
Apiaceae	*Eryngium heterophyllum* Engelm.	OS	P	N	H	NEA	---	---

GROUP/FAMILY	Taxa	Record source	Life Cycle	Residency status	Life form	Phyt. af.	Endemism	Conservation status
Apiaceae	*Eryngium lemmonii* J.M. Coult & Rose	CS	P	N	H	NEA	---	---
Apiaceae	*Eryngium globosum* Hemsl.	CS	P	N	H	NEA	EM	---
Apiaceae	*Ligusticum porteri* J.M. Coult & Rose	OS	P	N	G	NEA	---	IUCN-LC
Apiaceae	*Osmorhiza mexicana* Griseb.	CS	P	N	H	NEA	---	---
Apiaceae	*Prionosciadium thapsoides* (DC.) Mathias	CS	P	N	G	NEA	---	---
Apiaceae	*Tauschia edulis* (S.Watson) J.M.Coult. & Rose	CS	P	N	G	NEA	EM, ESMO	---
Asteraceae	*Acourtia wislizeni* (A.Gray) Reveal & R.M.King	OS	P	N	H	NEO	EM	---
Asteraceae	*Ageratina hyssopina* (A.Gray) R.M.King & H.Rob	OS	P	N	H	NEO	EM	---
Asteraceae	*Ageratina lemmonii* (B.L. Rob.) R.M. King & H. Rob.	OS	P	N	H	NEA	---	---
Asteraceae	*Ageratina rothrockii* (A.Gray) R.M.King & H.Rob.	CS	P	N	H	NEA	---	---
Asteraceae	*Artemisia ludoviciana* Nutt.	CS	P	N	H	NEA	---	---
Asteraceae	*Bidens aurea* (Aiton) Sherff	OS	P	N	H	NEO	---	---
Asteraceae	*Bidens pilosa* L.	CS	A	N	T	NEO	---	---
Asteraceae	*Bidens triplinervia* Kunth	CS	P	N	H	NEO	---	---
Asteraceae	*Carphochaete pringlei* (S. Watson) Grashoff ex B.L. Turner	OS	P	N	H	NEO	EM	---
Asteraceae	*Carphochaete wislizeni* A. Gray	OS	P	N	H	NEO	EM	---
Asteraceae	*Cirsium vergelense* G. L. Nesom	CS	P	N	H	NEA	EM, ESMO, ECH	---
Asteraceae	*Cosmos linearifolius* (Sch.Bip.) Hemsl.	CS	P	N	H	NEO	EM	---
Asteraceae	*Cosmos palmeri* B.L. Rob.	CS	P	N	H	NEO	EM	---
Asteraceae	*Cosmos parviflorus* (Jacq.) Pers.	CS	A	N	T	NEO	---	---
Asteraceae	*Dahlia sherffii* P.D.Sørensen	CS	P	N	G	NEO	EM	---
Asteraceae	*Erigeron caulinifolius* G.L.Nesom	CS	P	N	H	NEA	EM, ESMO, ECH, EMO	---
Asteraceae	*Erigeron divergens* Torr. & A. Gray	OS	A	N	T	NEA	---	---
Asteraceae	*Erigeron forreri* (Greene) Greene	OS	P	N	H	NEA	EM	---
Asteraceae	*Erigeron fraternus* Greene	OS	P	N	H	NEA	EM	---
Asteraceae	*Erigeron galeottii* (A.Gray ex Hemsl.) Greene	CS	P	N	H	NEA	EM	---
Asteraceae	*Erigeron griseus* (Greenm.) G.L.Nesom	OS	P	N	H	NEA	EM	---

GROUP/FAMILY	Taxa	Record source	Life Cycle	Residency status	Life form	Phyt. af.	Endemism	Conservation status
Asteraceae	*Erigeron macdonaldii* G.L. Nesom	OS	P	N	H	NEA	EM, ESMO, ECH, EMO	---
Asteraceae	*Erigeron mohinorensis* G.L. Nesom	CS	P	N	H	NEA	EM, ESMO, ECH, EMO	---
Asteraceae	*Erigeron oreophilus* Greenm.	OS	P	N	H	NEA	---	---
Asteraceae	*Erigeron rhizomactis* G.L. Nesom	CS	P	N	G	NEA	EM, ESMO	---
Asteraceae	*Galinsoga parviflora* Cav.	CS	A	N	T	NEO	---	---
Asteraceae	*Galinsoga subdiscoidea* Cronquist	OS	A	N	T	NEO	EM, ESMO	---
Asteraceae	*Galinsogeopsis montana* (A.M.Powell) Lichter-Marck	CS	A	N	T	NEO	EM, ESMO, ECH	---
Asteraceae	*Gamochaeta americana* (Mill.) Wedd.	CS	P	N	H	NEO	---	---
Asteraceae	*Guardiola rosei* B.L. Rob.	CS	P	N	H	NEO	EM, ESMO	---
Asteraceae	*Gutierrezia megalocephala* (Fernald) G.L.Nesom	CS	P	N	Ca	NEA	EM, ESMO	---
Asteraceae	*Heliomeris multiflora* Nutt. var. *macrocephala* Heiser	CS	P	N	H	NEA	---	---
Asteraceae	*Hieracium abscissum* Less.	CS	P	N	H	NEA	---	---
Asteraceae	*Hieracium fendleri* Sch.Bip. subsp. *fendleri* Sch. Bip.	CS	P	N	H	NEA	---	---
Asteraceae	*Iostephane heterophylla* Benth.	CS	P	N	H	NEO	EM	---
Asteraceae	*Leibnitzia lyrata* (Sch. Bip) G. L. Nesom	CS	P	N	H	NEA	---	---
Asteraceae	*Packera candidissima* (Greene) W. A. Weber & A. Löve	CS	P	N	H	NEA	EM	---
Asteraceae	*Packera scalaris* var. *scalaris* (Greene) C.Jeffrey	CS	P	N	H	NEA	EM, ESMO	---
Asteraceae	*Packera toluccana* (DC.) W.A.Weber & Á.Löve	CS	P	N	H	NEA	EM	---
Asteraceae	*Packera umbraculifera* (S.Watson) W.A.Weber & Á.Löve	CS	P	N	H	NEA	EM, ESMO	---
Asteraceae	*Pippenalia delphiniifolia* (Rydb.) McVaugh	CS	P	N	H	NEO	EM	---
Asteraceae	*Plectocephalus americanus* (Nutt.) D.Don	CS	A	N	T	NEA	---	---
Asteraceae	*Psacalium peltatum* Cass.	CS	P	N	G	NEO	EM	---
Asteraceae	*Pseudognaphalium viscosum* (Kunth) Anderb.	CS	A	N	T	NEO	---	---
Asteraceae	*Ratibida mexicana* W.M.Sharp	OS	P	N	H	NEA	EM	---
Asteraceae	*Roldana pennellii* H. Rob. & Brettell	CS	P	N	H	NEO	EM	---
Asteraceae	*Senecio chihuahuensis* S. Watson	CS	P	N	H	NEA	EM, ESMO	---

GROUP/FAMILY	Taxa	Record source	Life Cycle	Residency status	Life form	Phyt. af.	Endemism	Conservation status
Asteraceae	*Senecio mohinorensis* Greenm.	CS	P	N	H	NEA	EM, ESMO, ECH, EMO	---
Asteraceae	*Senecio multidentatus* Sch.Bip. ex Hemsl. var. *huachucanus* (A.Gray) T.M. Barkley	OS	P	N	H	NEA	---	---
Asteraceae	*Stevia plummerae* A.Gray	CS	P	N	H	NEA	---	---
Asteraceae	*Stevia serrata* Cav.	CS	P	N	H	NEO	---	---
Asteraceae	*Tagetes micrantha* Cav.	CS	A	N	T	NEO	---	---
Asteraceae	*Tagetes lucida* Cav.	OS	P	N	H	NEO	---	---
Asteraceae	*Tagetes palmeri* A. Gray	OS	P	N	H	NEO	EM	---
Asteraceae	*Taraxacum officinale* F.H.Wigg.	CS	P	I	H	-----	---	IUCN-LC
Asteraceae	*Verbesina longifolia* A. Gray	CS	P	N	H	NEO	---	---
Asteraceae	*Verbesina scotiodonta* S.F.Blake	CS	P	N	H	NEO	EM, ESMO, ECH, EMO	---
Begoniaceae	*Begonia gracilis* Kunth	CS	P	N	G	NEO	---	---
Betulaceae	*Alnus oblongifolia* Torr.	CS	P	N	F	NEA	---	IUCN-LC
Boraginaceae	*Lithospermum thurberi* (A.Gray) J.I.Cohen	OS	P	N	H	NEA	---	---
Boraginaceae	*Phacelia platycarpa* (Cav.) Spreng.	CS	P	N	H	NEA	---	---
Brassicaceae	*Brassica rapa* L.	CS	A	I	T	-----	---	---
Brassicaceae	*Draba helleriana* Greene	CS	P	N	H	NEA	EM	---
Brassicaceae	*Noccaea fendleri* (A. Gray) Holub	CS	P	N	H	NEA	---	---
Brassicaceae	*Pennellia longifolia* (Benth.) Rollins	CS	P	N	H	NEA	---	---
Brassicaceae	*Romanschulzia correllii* Rollins	OS	P	N	H	NEO	EM, ESMO, ECH, EMO	---
Cactaceae	*Mammillaria senilis* Lodd. Ex Salm-Dyck	CS	P	N	Ca	NEO	EM, ESMO	NOM-A, IUCN-LC, CITES-II
Cactaceae	*Opuntia robusta* H.L. Wendl. ex Pfeiff.	CS	P	N	F	NEO	---	CITES-II
Campanulaceae	*Lobelia ehrenbergii* (Vatke) Kuntze	CS	P	N	H	NEO	EM	---
Caprifoliaceae	*Lonicera pilosa* (Kunth) Steud.	CS	P	N	F	NEA	EM	---
Caprifoliaceae	*Symphoricarpos oreophilus* A. Gray	OS	P	N	F	NEA	---	---
Caprifoliaceae	*Valeriana deltoidea* F.G. Mey.	CS	P	N	H	NEO	EM	---

GROUP/FAMILY	Taxa	Record source	Life Cycle	Residency status	Life form	Phyt. af.	Endemism	Conservation status
Caprifoliaceae	*Valeriana sorbifolia* Kunth	CS	P	N	H	NEO	---	---
Caryophyllaceae	*Arenaria lanuginosa (Michx.) Rohrb.*	CS	P	N	H	NEA	---	IUCN-LC
Caryophyllaceae	*Cerastium madrense* S. Watson	CS	P	N	H	NEA	EM, ESMO	---
Caryophyllaceae	*Cerastium nutans* Raf.	CS	A	N	T	NEA	---	---
Caryophyllaceae	*Drymaria effusa* A. Gray	CS	A	N	T	NEO	---	---
Caryophyllaceae	*Drymaria villosa* Schltdl. & Cham.	CS	A	N	T	NEO	---	---
Caryophyllaceae	*Silene laciniata* subsp. *greggii* (A. Gray) C. L. Hitchc. & Maguire	CS	P	N	H	NEA	---	---
Caryophyllaceae	*Silene scouleri* subsp. *pringlei* (S. Watson) C.L. Hitchc. & Maguire	OS	P	N	H	NEA	---	---
Convolvulaceae	*Cuscuta odontolepis* Engelm.	CS	A	N	T	NEO	---	---
Convolvulaceae	*Ipomoea capillacea* (Kunth) G. Don	CS	P	N	G	NEO	---	---
Convolvulaceae	*Ipomoea pubescens* Lam.	CS	P	N	G	NEO	---	---
Cornaceae	*Cornus sericea* L.	CS	P	N	F	WD	---	IUCN-LC
Crassulaceae	*Echeveria affinis* E. Walther	CS	P	N	Ca	NEO	EM, ESMO	---
Crassulaceae	*Sedum chihuahuense* S. Watson	CS	A	N	T	NEO	EM	---
Crassulaceae	*Sedum jaliscanum* S. Watson	OS	A	N	T	NEO	EM	---
Crassulaceae	*Sedum pringlei* S. Watson	OS	P	N	H	NEO	EM	---
Crassulaceae	*Sedum sinforosanum* J. Reyes, Etter & Kristen	CS	P	N	H	NEO	EM, ESMO, ECH	---
Crassulaceae	*Sedum stelliforme* S.Watson	CS	P	N	H	NEO	---	---
Crassulaceae	*Villadia pringlei* Rose	CS	P	N	Ca	NEO	EM	---
Cucurbitaceae	*Cucurbita foetidissima* Kunth	CS	P	N	G	NEA	---	IUCN-LC
Cucurbitaceae	*Microsechium palmatum* (Ser.) Cogn.	CS	P	N	G	NEO	---	---
Ericaceae	*Arbutus bicolor* S. González, M. González & P.D. Sørensen	CS	P	N	F	NEA	EM	IUCN-LC
Ericaceae	*Arbutus mollis* Kunth	CS	P	N	F	NEA	EM	---
Ericaceae	*Arbutus occidentalis* McVaugh & Rosatti	CS	P	N	F	NEA	EM	---
Ericaceae	*Arbutus tessellata* P.D. Sorensen	CS	P	N	F	NEA	EM	IUCN-LC
Ericaceae	*Arbutus xalapensis* Kunth	CS	P	N	F	NEA	---	IUCN-LC
Ericaceae	*Arctostaphylos pungens* Kunth	CS	P	N	F	NEA	---	IUCN-LC

GROUP/FAMILY	Taxa	Record source	Life Cycle	Residency status	Life form	Phyt. af.	Endemism	Conservation status
Ericaceae	*Chimaphila maculata* (L.) Pursh	CS	P	N	H	NEA	---	---
Ericaceae	*Chimaphila umbellata* (L.) W.P.C. Barton	CS	P	N	H	WD	---	---
Ericaceae	*Comarostaphylis polifolia* (Kunth) Zucc. ex Klotzsch	CS	P	N	F	NEA	EM	---
Ericaceae	*Monotropa hypopitys* L.	CS	P	N	H	WD	---	NOM-Pr
Ericaceae	*Orthilia secunda* (L.) House	CS	P	N	H	WD	---	---
Ericaceae	*Vaccinium caespitosum* Michx.	CS	P	N	H	NEA	---	---
Euphorbiaceae	*Euphorbia furcillata* Kunth	CS	P	N	H	NEO	---	---
Euphorbiaceae	*Euphorbia sphaerorhiza* Benth.	OS	P	N	G	NEO	EM	---
Fabaceae	*Acmispon oroboides* (Kunth) Brouillet	CS	P	N	H	NEA	---	---
Fabaceae	*Amicia zygomeris* DC.	OS	P	N	H	NEO	EM	---
Fabaceae	*Astragalus vaccarum* A. Gray	OS	P	N	H	NEA	---	---
Fabaceae	*Dalea filiformis* A. Gray	CS	A	N	T	NEO	---	---
Fabaceae	*Dalea nelsonii* (Rydb.) Barneby	CS	P	N	H	NEO	EM, ESMO	---
Fabaceae	*Dalea obreniformis* (Rydb.) Barneby	CS	A	N	T	NEO	EM	---
Fabaceae	*Hosackia alamosana* Rose	CS	P	N	H	NEA	---	---
Fabaceae	*Hosackia repens* G. Don	CS	P	N	H	NEA	EM	---
Fabaceae	*Lupinus chihuahuensis* S. Watson	CS	P	N	H	NEA	EM, ESMO	---
Fabaceae	*Lupinus howardii* M.E. Jones	CS	P	N	H	NEA	---	---
Fabaceae	*Lupinus huachucanus* M.E. Jones	CS	P	N	H	NEA	---	---
Fabaceae	*Lupinus lesueurii* Standl.	CS	P	N	H	NEA	EM, ESMO, ECH	---
Fabaceae	*Lupinus montanus* Kunth subsp. *glabrior* (S. Watson) D. B. Dumm & Harmon	CS	P	N	H	NEA	EM, ESMO	---
Fabaceae	*Phaseolus jaliscanus* Piper	CS	P	N	G	NEO	EM	IUCN-LC
Fabaceae	*Phaseolus parvulus* Greene	CS	P	N	G	NEO	---	IUCN-LC
Fabaceae	*Trifolium amabile* Kunth	CS	P	N	H	NEA	---	IUCN-LC
Fabaceae	*Vicia leucophaea* Greene	OS	A	N	T	NEA	---	IUCN-LC
Fabaceae	*Quercus arizonica* Sarg.	CS	P	N	F	NEA	---	IUCN-LC
Fagaceae	*Quercus crassifolia* Bonpl.	OS	P	N	F	NEA	---	IUCN-LC
Fagaceae	*Quercus mcvaughii* Spellenb.	CS	P	N	F	NEA	EM	IUCN-NT

GROUP/FAMILY	Taxa	Record source	Life Cycle	Residency status	Life form	Phyt. af.	Endemism	Conservation status
Fagaceae	*Quercus rugosa* Née	CS	P	N	F	NEA	---	IUCN-LC
Fagaceae	*Quercus sideroxyla* Bonpl.	CS	P	N	F	NEA	EM	IUCN-LC
Garryaceae	*Garrya laurifolia* Benth.	CS	P	N	F	NEA	---	IUCN-LC
Gentianaceae	*Gentianella amarella* (L.) Börner	CS	A	N	T	WD	---	---
Gentianaceae	*Gentianopsis detonsa* (Rottb.) Ma var. *superba* (Greene) Villarreal et A.E. Estrada	CS	A	N	T	NEA	---	---
Gentianaceae	*Halenia recurva* (Sm.) C.K. Allen	CS	A	N	T	NEO	---	---
Geraniaceae	*Erodium cicutarium* (L.) L'Hér.	CS	A	I	T	-----	---	---
Geraniaceae	*Geranium caespitosum* E. James	OS	P	N	H	NEA	---	---
Geraniaceae	*Geranium richardsonii* Fisch. & Trautv.	CS	P	N	H	NEA	---	---
Geraniaceae	*Geranium seemannii* Peyr. *subsp. seemannii*	OS	P	N	H	NEA	---	---
Geraniaceae	*Geranium wislizeni* S. Watson	CS	P	N	H	NEA	---	---
Grossulariaceae	*Ribes ceriferum* Coville & Rose	CS	P	N	F	NEA	EM	---
Grossulariaceae	*Ribes madrense* Coville & Rose	CS	P	N	F	NEA	---	---
Hypericaceae	*Hypericum formosum* Kunth	CS	P	N	H	NEO	---	---
Lamiaceae	*Agastache coccinea* (Greene) Lint & Epling	CS	P	N	H	NEO	EM, ESMO	---
Lamiaceae	*Lepechinia caulescens* (Ortega) Epling	CS	P	N	H	NEO	---	---
Lamiaceae	*Lepechinia schiedeana* (Schltdl.) Vatke	CS	P	N	H	NEO	---	---
Lamiaceae	*Monarda citriodora* Cerv. Ex Lag.	CS	A	N	T	NEA	---	---
Lamiaceae	*Prunella vulgaris* L.	CS	P	N	H	WD	---	IUCN-LC
Lamiaceae	*Salvia clinopodioides* Kunth	CS	P	N	H	NEO	EM	---
Lamiaceae	*Salvia decora* Epling	CS	P	N	H	NEO	EM	---
Lamiaceae	*Salvia lavanduloides* Kunth	CS	P	N	H	NEO	---	---
Lamiaceae	*Salvia microphylla* Kunth	OS	P	N	Ca	NEO	---	---
Lamiaceae	*Salvia reginae* J.G. González & J.H. Vega	CS	P	N	G	NEO	EM, ESMO, ECH	---
Lamiaceae	*Stachys agraria* Schltdl. & Cham.	CS	A	N	T	NEO	---	---
Lamiaceae	*Stachys coccinea* Ortega	CS	P	N	H	NEO	---	---
Lamiaceae	*Stachys mohinora* B.L. Turner	CS	P	N	H	NEO	EM, ESMO, ECH, EMO	---
Lentibulariaceae	*Pinguicula parvifolia* B.L. Rob.	CS	P	N	H	NEO	EM	---

GROUP/FAMILY	Taxa	Record source	Life Cycle	Residency status	Life form	Phyt. af.	Endemism	Conservation status
Linaceae	*Linum pringlei* S. Watson	OS	A	N	T	NEA	EM	---
Montiaceae	*Claytonia perfoliata* Donn ex Willd.	CS	A	N	T	NEA	---	---
Onagraceae	*Lopezia gracilis* S. Watson	CS	P	N	H	NEO	EM, ESMO	---
Onagraceae	*Oenothera luciae-julianiae* W.L. Wagner	CS	P	N	H	NEA	EM	---
Onagraceae	*Oenothera pubescens* Willd. Ex Spreng.	CS	P	N	H	NEA	---	---
Onagraceae	*Oenothera rosea* L'Hér. Ex Aiton	CS	P	N	H	WD	---	---
Orobanchaceae	*Castilleja nelsonii* Eastw.	OS	P	N	H	NEA	---	---
Orobanchaceae	*Castilleja patriotica* Fernald	CS	P	N	H	NEA	---	---
Orobanchaceae	*Escobedia peduncularis* Pennell	OS	P	N	H	NEA	EM	---
Orobanchaceae	*Pedicularis angustifolia* Benth.	CS	P	N	H	NEA	---	---
Orobanchaceae	*Pedicularis chihuahuensis* G.L. Nesom	CS	P	N	H	NEA	EM, ESMO, ECH	---
Orobanchaceae	*Seymeria bipinnatisecta* Seem.	CS	A	N	T	NEA	---	---
Oxalidaceae	*Oxalis alpina* (Rose) Rose ex R. Knuth	CS	P	N	G	NEO	---	---
Oxalidaceae	*Oxalis corniculata* L.	CS	P	I	H	-----	---	---
Oxalidaceae	*Oxalis decaphylla* Kunth	OS	P	N	G	NEO	---	---
Oxalidaceae	*Oxalis drummondii* A. Gray	CS	P	N	G	NEO	---	---
Papaveraceae	*Corydalis aurea* subsp. *aurea* Willd.	OS	A	N	T	NEA	---	---
Phrymaceae	*Erythranthe glabrata* (Kunth) G.L. Nesom	CS	P	N	H	NEO	---	---
Phytolaccaceae	*Phytolacca octandra* L.	CS	A	I	T	-----	---	---
Plantaginaceae	*Penstemon barbatus* (Cav.) Roth	CS	P	N	H	NEA	---	---
Plantaginaceae	*Penstemon campanulatus* (Cav.) Willd.	CS	P	N	H	NEA	---	---
Plantaginaceae	*Penstemon mohinoranus* Straw	OS	P	N	H	NEA	EM, ESMO, ECH, EMO	---
Plantaginaceae	*Penstemon wislizeni* (A. Gray) Straw	OS	P	N	H	NEA	EM	---
Plantaginaceae	*Plantago australis* Lam.	CS	P	N	H	NEA	---	---
Plantaginaceae	*Plantago linearis* Kunth	OS	A	N	T	NEA	---	---
Plantaginaceae	*Sibthorpia repens* (Mutis ex L.) Kuntze	CS	P	N	H	NEA	---	---
Plantaginaceae	*Veronica mexicana* S. Watson	OS	P	N	H	NEO	EM	---
Plantaginaceae	*Veronica peregrina* L.	OS	A	N	T	WD		IUCN-LC

GROUP/FAMILY	Taxa	Record source	Life Cycle	Residency status	Life form	Phyt. af.	Endemism	Conservation status
Polemoniaceae	*Polemonium glabrum* J.F. Davidson	CS	P	N	H	NEA	EM, ESMO, ECH, EMO	---
Polemoniaceae	*Polemonium grandiflorum* Benth.	CS	P	N	H	NEA	EM	---
Polygonaceae	*Rumex acetosella* L.	CS	P	I	H	-----	---	---
Polygonaceae	*Rumex obtusifolius* L.	CS	P	I	H	-----	---	---
Primulaceae	*Primula pauciflora* (Greene) A.R. Mast & Reveal	OS	P	N	H	NEA	---	---
Primulaceae	*Primula rusbyi* Greene	OS	P	N	H	NEA	---	---
Primulaceae	*Primula standleyana* A.R.Mast & Reveal	CS	P	N	H	NEA	---	---
Ranunculaceae	*Actaea rubra* (Aiton) Willd.	CS	P	N	H	NEA	---	---
Ranunculaceae	*Aquilegia skinneri* Hook. f.	CS	P	N	H	NEA	EM	---
Ranunculaceae	*Delphinium subscandens* Ewan	CS	P	N	H	NEA	EM	---
Ranunculaceae	*Ranunculus gentryanus* L.D. Benson	CS	P	N	H	NEA	EM, ESMO, ECH	---
Ranunculaceae	*Thalictrum grandifolium* S. Watson	CS	P	N	H	NEA	EM	---
Rhamnaceae	*Ceanothus buxifolius* Willd. ex Schult. & Schult.f.	CS	P	N	F	NEA	EM	---
Rhamnaceae	*Ceanothus caeruleus* Lag.	CS	P	N	F	NEA	---	IUCN-LC
Rosaceae	*Agrimonia striata* Michx.	OS	P	N	H	NEA	---	---
Rosaceae	*Alchemilla aphanoides* (Mutis ex L.f.) Rothm.	OS	P	N	H	NEA	---	---
Rosaceae	*Alchemilla procumbens* (Rose)	CS	P	N	H	NEA	---	---
Rosaceae	*Fragaria vesca* L.	CS	P	N	H	WD	---	---
Rosaceae	*Holodiscus discolor* (Pursh) Maxim.	CS	P	N	F	NEA	---	---
Rosaceae	*Potentilla exsul* Standl.	CS	P	N	H	NEA	EM, ESMO, ECH	---
Rosaceae	*Potentilla mexiae* Standl.	CS	P	N	H	NEA	EM	---
Rosaceae	*Potentilla thurberi* A. Gray	CS	P	N	H	NEA	---	---
Rosaceae	*Prunus serotina* var. *rufula* (Wooton & Standl.) McVaugh	CS	P	N	F	NEA	---	---
Rosaceae	*Prunus serotina* var. *salicifolia* (Kunth) Koehne	CS	P	N	F	NEA	---	---
Rosaceae	*Rubus idaeus* subsp. *strigosus* L. (Michx.) Focke	OS	P	N	F	WD	---	---
Rosaceae	*Rubus pringlei* Rydb.	CS	P	N	F	NEA	---	---
Rosaceae	*Rubus pumilus* Focke	CS	P	N	H	NEA	EM	---

GROUP/FAMILY	Taxa	Record source	Life Cycle	Residency status	Life form	Phyt. af.	Endemism	Conservation status
Rubiaceae	*Bouvardia ternifolia* (Cav.) Schltdl.	CS	P	N	F	NEO	---	---
Rubiaceae	*Crusea longiflora* (Roem. & Schult.) W.R. Anderson	CS	A	N	T	NEO	---	---
Rubiaceae	*Galium mexicanum subsp. asperrimum* (A. Gray) Dempster	CS	P	N	H	NEA	---	---
Rubiaceae	*Galium uncinulatum* DC.	CS	P	N	H	NEA	---	---
Salicaceae	*Populus tremuloides* Michx.	CS	P	N	F	NEA	---	IUCN-LC
Salicaceae	*Salix nigra* Marshall	CS	P	N	F	NEA	---	IUCN-LC
Salicaceae	*Salix paradoxa* Kunth	OS	P	N	F	NEA	EM	IUCN-LC
Santalaceae	*Arceuthobium abietinum* (Engelm.) Munz	OS	P	N	Hp	NEA	---	---
Santalaceae	*Arceuthobium blumeri* A. Nelson	CS	P	N	Hp	NEA	---	---
Santalaceae	*Arceuthobium douglasii* Engelm.	OS	P	N	Hp	NEA	---	---
Santalaceae	*Arceuthobium globosum* subsp. *globosum* Hawksw. & Wiens	OS	P	N	Hp	NEA	---	---
Santalaceae	*Arceuthobium globosum* subsp. *grandicaule* Hawksw. & Wiens	OS	P	N	Hp	NEA	---	---
Santalaceae	*Arceuthobium vaginatum* (Humb. & Bonpl. ex Willd.) J.Presl	OS	P	N	Hp	NEA	---	---
Santalaceae	*Phoradendron longifolium* Eichler ex Trel.	CS	P	N	Hp	NEO	EM	---
Saxifragaceae	*Heuchera mexicana var. mexicana*	CS	P	N	H	NEA	---	---
Saxifragaceae	*Micranthes eriophora* (S. Watson) Small	CS	P	N	H	NEA	---	---
Scrophulariaceae	*Buddleja parviflora* Kunth	CS	P	N	F	NEO	---	IUCN-LC
Solanaceae	*Datura wrightii* Regel	CS	A	N	T	NEA	---	---
Solanaceae	*Solanum demissum* Benth.	CS	P	N	G	NEA	---	IUCN-LC
Solanaceae	*Solanum stoloniferum* Schltdl.	CS	P	N	G	NEA	---	IUCN-LC
Urticaceae	*Urtica gracilis* Aiton	CS	P	N	H	NEA	---	---
Verbenaceae	*Glandularia bipinnatifida* Nutt.	CS	P	N	H	NEO	---	---
Verbenaceae	*Verbena carolina* L.	CS	P	N	H	NEO	---	---
Verbenaceae	*Verbena menthifolia* Benth.	CS	P	N	H	NEO	---	---
Viburnaceae	*Sambucus cerulea* Raf.	CS	P	N	F	NEA	---	---
Violaceae	*Viola canadensis* L.	CS	P	N	H	NEA	---	---
Violaceae	*Viola umbraticola* Kunth	CS	P	N	H	NEA	---	---

GROUP/FAMILY	Taxa	Record source	Life Cycle	Residency status	Life form	Phyt. af.	Endemism	Conservation status
Vitaceae	*Vitis arizonica* Engelm.	CS	P	N	F	NEA	---	---
